# Personality Traits of Suicidality Are Associated with Premenstrual Syndrome and Premenstrual Dysphoric Disorder in a Suicidal Women Sample

**DOI:** 10.1371/journal.pone.0148653

**Published:** 2016-02-10

**Authors:** Déborah Ducasse, Isabelle Jaussent, Emilie Olié, Sébastien Guillaume, Jorge Lopez-Castroman, Philippe Courtet

**Affiliations:** 1 Department of Psychiatric Emergency & Acute Care, Lapeyronie Hospital, CHU Montpellier, Montpellier, France; 2 Inserm U1061, University of Montpellier UM1, Montpellier, France; 3 Fondamental Foundation, Créteil, France; Central Institute of Mental Health, GERMANY

## Abstract

**Objective:**

Both Premenstrual Syndrome (PMS) and Premenstrual Dysphoric Disorder (PMDD) might increase the risk of suicidal behavior. The aim of this study was to assess the relationship between personality dimensions specifically involved in suicidal vulnerability and PMS/PMDD.

**Method:**

We collected data from 232 women consecutively hospitalized after a suicide attempt. We examined the relationship between impulsivity, aggressiveness/hostility, hopelessness, trait anger, affect intensity, emotional lability, and PMS/PMDD. Notably, we created an algorithm from the shortened Premenstrual Assessment form in order to assess PMDD status.

**Results:**

The proportions of PMS and PMDD among female suicide attempters were 50% and 23% respectively. Women with PMS or PMDD were more likely to endorse most of these personality traits to than those without even after controlling for potential confounders. We found an impulsive-aggressive pattern of personality in women with PMS or PMDD, independently from the time of the menstrual cycle. Interestingly, trait anger remained associated with both PMS and PMDD independently of every other personality traits. The higher the anger level, the higher the risk was to suffer from both PMS and PMDD.

**Conclusions:**

This study demonstrates a strong, independent association between PMS/PMDD and trait anger among a representative sample of female suicide attempters. It is of major interest for clinicians in view of addressing a substantial public health problem among women of reproductive age.

## Introduction

The array of premenstrual complaints can be grouped into three categories: 1) Minor premenstrual symptoms that do not cause functional impairments and are minimally distressing; 2) Premenstrual syndrome (PMS) that includes distressing symptoms (i.e. moderate to severe symptomatology); 3) Premenstrual dysphoric disorder (PMDD), the most severe form of PMS, which is a codified condition that adheres to diagnostic criteria outlined in the Diagnostic and Statistical Manual of Mental Disorders (DSM-5) [1]

Premenstrual syndrome (PMS) is a common disorder of young and middle-aged women, characterized by emotional and physical symptoms that consistently recur in a cyclic manner during the luteal phase of the menstrual cycle and typically disappear with menopause [2]. Although up to 90% of women of reproductive age experience minor premenstrual symptoms [3], approximately 20% of them experience PMS that substantially impair their daily life [2, 4]. In 2000, the American College of Obstetricians and Gynecologists (ACOG) published a practice bulletin on PMS containing diagnostic criteria: at least one of the following affective and somatic symptoms during the five days before menses in each of three prior menstrual cycles: depression, angry outburst, anxiety, irritability, confusion, social withdrawal (for the affective symptoms); breast tenderness, abdominal bloating, headache, swelling of extremities (for the somatic symptoms) [5, 6]. Symptoms recede within 4 days of the onset of menstruation and do not recur until at least day 13 of the menstrual cycle [6, 7]. Patients suffer from identifiable dysfunction in social or economic performance [6]. Indeed, decreased work productivity, increased work absenteeism and seeking healthcare support were reported in women with PMS vs. women not affected by PMS, resulting in considerable economic losses [8]. Interestingly, in clinical samples of women affected by PMS, 31% of them matched the criteria for mood disorders [9], and 25% for comorbid anxiety disorder [9, 10].

The inclusion of Premenstrual Dysphoric Disorder (PMDD) in the DSM 5 [1] as a distinct category remains controversial [2, 5]. See supplementary material for PMDD diagnostic criteria according to the DSM-5. Although the range of possible symptoms needed for a diagnosis is the same in both PMDD and PMS, PMS diagnosis requires just one of these symptoms (not necessarily affective), while PMDD diagnosis requires at least five and one of them being a mood symptom (irritability, affect lability, depressed mood, anxiety). Indeed, PMDD can be seen as a severe form of PMS [11], affecting 2–5% of premenopausal women [12]. The burden of PMS/PMDD is high, with an estimated disability weight for severe PMS at around 0.5 according to the Global Burden of Disease model [13], thus approaching that of unipolar major depression [2]. This 0.5 disability weight can be translated into 1400 days or 3.83 years of disability (DALY) for every woman. According to the US 2000 census, there were 75,580,000 women aged 14–51 and among them at least 3,779,000 (5% prevalence) met PMDD criteria. Therefore, PMDD could account for nearly 15 million DALYs in the US alone.

Compared to women without premenstrual symptoms, women with PMDD seem to be two or three times more likely to report lifetime suicidal ideation [14, 15], suicidal plans and attempts [15], independently of psychiatric comorbidities (Major Depressive Disorder, anxiety disorder and substance use disorder) and demographic characteristics. However, only one study assessed the association between PMS and suicidal behavior [16]. This cross-sectional study, conducted on 2411 adolescent females in secondary school, found that females with PMS were more likely to endorse suicidal ideas, but not more likely to attempt suicide.

According to the “vulnerability x stress” model [17], sensitive subjects will be prone to suicidal behaviors when confronted to psychiatric or social stressors. Suicidal vulnerability involves personality traits, such as impulsive aggression, hostility and anger [18], hopelessness and pessimism [19], neuroticism, and harm avoidance [20]. Interestingly, some personality traits might be associated with the likeliness to suffer from PMS/PMDD. Berlin et al [21] found that women with PMS scored higher than controls on histrionic, obsessive-compulsive, self-defeating and dependent subscales of the Personality Disorder Questionnaire-Revised, independently of the menstrual phase. Similarly, Sasson et al. [22] reported more personality disorders in women with PMS/PMDD compared to women without (according to the Structured Clinical Interview for DSM IV). Finally, several studies found a significant association between neuroticism and PMS/PMDD [23, 24].

Finally, even if suicidal behavior and PMS/PMDD are common disorders among women, mechanisms underlying this association remain unknown. The objective of this study was to examine the relationship of PMS and PMDD with personality dimensions involved in suicidal vulnerability: impulsivity, aggressiveness/hostility, hopelessness, trait anger, affect intensity and lability.

## Methods

### 2.1. Participants

Participants were recruited as part of a cohort of Caucasian suicide attempters, consecutively admitted in a psychiatric inpatient unit of the Montpellier University Hospital. We selected French-speaking non-menopausal women over the age of 18. A suicide attempt was defined as a self-damaging act carried out with some intent to die, distinguished from other self-destructive behaviors such as self-mutilation, non-compliance with medical treatment in severely ill individuals, and the use of substances such as alcohol and tobacco [25]. Violent suicide attempts were classified using the criteria of Asberg et al [26]. According to these criteria, a suicide attempt was defined as violent when the method of suicide attempt was hanging, use of firearms, jumping from heights, several deep cuts, car crash, burning, gas poisoning, drowning, electrocution, or jumping under a train. Suicide attempts were defined as nonviolent when the method was drug overdose or superficial wrist cutting. Cutting was considered violent when it required surgical treatment, i.e. deep sutures to close an open wound. An overdose requiring hospitalization in an intensive care unit was considered a serious suicide attempt. Consequently, non-serious, nonviolent suicide attempts consisted of drug overdoses or superficial wrist cutting that did not require treatment in an intensive care unit. Trained senior psychiatrists and psychologists performed the clinical assessments. The local research ethics committee approved this study (CPP Sud Mediterranée IV, CHU Montpellier, France) and all patients signed a consent form.

### 2.2. Clinical assessment

#### PMS and PMDD status

PMS status was evaluated using the shortened version of the Premenstrual Assessment Form (PAF). It is a validated self-assessing questionnaire identifying changes in mood, behavior and physical well-being during the premenstrual period (from 1 to 14 days prior to the onset of menses). Patients self-reported actual changes when they were in their premenstrual period, or common changes that occurred in their past three menstrual cycles. This questionnaire contains 33 items [27]. Cronbach’s Alpha for internal reliability revealed a high alpha (0.98). A score of 115 or above suggests moderate to severe symptoms of PMS [27, 28]. As a result we used this cut-off to evaluate PMS diagnosis.

PMDD status, a severe form of PMS characterized by more restrictive criteria and a predominance of emotional and behavioral symptoms [29, 30], was assessed using DSM 5 items from the PAF-shortened form [27, 28]. See supplementary material for details about these criteria.

#### Personality dimensions

Six personality dimensions previously involved in suicidal vulnerability were assessed [31–33]:

- Impulsivity was assessed using the Barratt Impulsivity Scale—tenth version (BIS 10) [34]. This scale contains 34 items rated from 0 to 4. It is subdivided into three different subscales: non-planning, motor, and cognitive impulsivity.
- Aggressiveness/hostility was assessed using the Buss-Durkee Hostility Scale (BDHI) [35]. It is a 75 true-false items inventory consisting of the following subscales: Assault, Indirect Hostility, Irritability, Negativism, Resentment, Suspicion, Verbal Hostility, and Guilt. The total score consists in the sum of all subscale scores and ranges from 1 to 75.
- Anger was assessed using the Spielberger State-Trait Anger Expression Inventory (STAXI) [36, 37]. It is a self-assessment scale with 44 items designed to evaluate a person’s anger level. Each item is essentially a statement to describe one’s emotions when feeling angry. Each item is assessed by a four-point scale (1 = no anger; 4 = maximum anger). We focused on the trait anger dimension in the present study.
- Hopelessness was assessed using the Beck Hopelessness Scale (BHS) [38, 39]. It is a self-report scale consisting of 20 true-false statements. The score ranges from 0 to 20.
- Affect intensity was assessed using the 44 items of the Affect Intensity Measure Scale [40]. This self-report scale examines affect intensity via the responses given to emotional reactions to typical life events. Each item is rated on a six-point scale.
- Affect lability was assessed using the Affect Lability Scale (ALS)-54 items [41]. This self-report scale assesses changeability or shifts in affect. Items are scored on a four-point scale ranging from 1 = not like me at all, to 4 = very much like me.

#### Other clinical and demographic variables

Axis-I DSM-IV diagnoses were assessed using the Diagnostic Interview for Genetics Studies (DIGS) [42] or the Mini International Neuropsychiatric Interview (MINI] [43]. We used either DIGS or MINI depending on the assessment but not both diagnostic interviews at the same time. We used this procedure to simplify the diagnostic assessment in patients who made serious or violent attempts (DIGS is more time-consuming). Lifetime diagnoses were determined using a best-estimate procedure: the psychiatrist in charge of the patient’s care established the diagnosis based on the MINI or DIGS interview, medical records and, when available, information from relatives [44]. Psychiatric comorbidities included lifetime diagnostic of depression and bipolar disorder, anxiety disorders, substance/alcohol use disorder, current depression severity, and tobacco consumption. Current depressive symptom levels were assessed using the Beck Depression Inventory-II (BDI-II) [45].

Sociodemographic variables were age, marital status (unmarried/married), present occupation (unemployed/employed), educational attainment (< 12 years of study /≥12 years of study), and hormonal birth control.

## Statistical Analysis

AAssociations between patients' characteristics, personality traits and PMS were quantified with odds ratios (OR) and their 95% confidence intervals (CI). Sociodemographic and clinical variables associated with PMS (at p<0.15) were included in logistic regression models as potential confounders to estimate the adjusted OR for personality traits. Personality scores were divided into tertiles because the linearity assumption was rejected for most of them. In order to determine the personality traits independently associated with the presence of severe PMS, personality traits associated with PMS at p<0.15 were entered in a logistic regression model, as well as sociodemographic and clinical variables associated with PMS at p<0.15, entered as potential confounders. Only the total scores were entered in the model due to the colinearity of the dimensions. The same methodology was used to study PMDD. Significance level was set at p<0.05. Analyses were performed using SAS statistical software (version 9.4; SAS Inc, Cary, NC).

## Results

### 4.1. Sample description

The study sample included 232 female suicide attempters. The median age was 33.80 years [range: 18.05–53.55]. Most patients had an educational attainment > 12 years (i.e. above High School Diploma) (71.1%). Approximately, one-third of patients were married (36.2%), and one-half was employed (55.6%).

Most patients (66.2%) had a BDI total score > 15. Concerning the history of suicide attempts, 12.1% of patients had made a violent attempt, 19.0% a severe attempt and 39.7% had made 3 attempts or more. The most frequent lifetime diagnoses were anxiety disorders (78.9%) and major depression (72.8%), followed by lifetime bipolar disorder (24.1%), alcohol abuse or dependence (28.0%) and substance abuse or dependence (18.9%). Finally, 56.0% took hormonal birth control. In our sample, 50% suffered from PMS (severe or not) (n = 116). Among these PMS patients, 44.7% (n = 51) suffered from PMDD.

### 4.2. Personality traits and PMS

Baseline characteristics of the sample according to the presence or absence of PMS are described in Table 1. Patients with PMS (PAF ≥115) were more likely to have a low educational attainment (p = 0.007), with less lifetime MDD (p = 0.006) but more bipolar disorder (p = 0.007), anxiety disorders (p = 0.04), and severe current depression (p = 0.008) than patients without PMS. Subsequent analyses were thus adjusted for these factors, and for the number of suicide attempts (p = 0.11). Others demographic covariates (age, marital status, present occupation, hormonal birth control) and psychiatric comorbidities (substance/alcohol use disorder and tobacco use, violence or severity of attempts) were not significantly associated with PMS. In regards to the different phases of the menstrual cycle, no significant difference was evidenced between women with PMS and those without (p = 0.54).

**Table 1:** Sociodemographic and clinical characteristics of patients according to PMS or PMDD.

	PMS						PMDD					
	PAF<115 N = 116		PAF≥115 N = 116				No N = 177		Yes N = 53			
	n	%	n	%	OR [95%CI]	p	n	%	n	%	OR [95%CI]	p
Age(years) *OR for 10-year increments*	34.18 (18.37–53.54)		33.35 (18.05–52.61)		0.96 [0.74–1.24]	0.75	35.35 (18.37–53.54)		29.55 (18.05–52.61)		0.75 [0.55–1.02]	0.07
Married	40	34.48	44	37.93	1.16 [0.68;1.98]	0.59	64	36.16	19	35.85	0.99 [0.52;1.87]	0.97
Employed	64	55.17	65	56.03	1.04 [0.62;1.74]	0.89	102	57.63	26	49.06	0.71 [0.38;1.31]	0.27
Educational level ≥12 years	92	79.31	73	62.93	0.44 [0.25;0.80]	0.007	133	75.14	32	60.38	0.50 [0.26;0.96]	0.04
Current tobacco smoking	67	57.76	67	57.76	1.00 [0.59;1.68]	0.99	100	56.50	32	60.38	1.17 [0.63;2.19]	0.62
Hormonal birth control	68	58.62	62	53.45	0.81 [0.48;1.36]	0.43	96	54.24	33	62.26	1.39 [0.74;2.61]	0.30
Lifetime MDD	94	81.03	75	64.66	0.43 [0.23;0.78]	0.006	132	74.58	35	66.04	0.66 [0.34;1.28]	0.22
Bipolar disorder	19	16.38	37	31.90	2.39 [1.28;4.48]	0.007	40	22.60	16	30.19	1.48 [0.75;2.94]	0.26
Anxiety disorders	85	73.28	98	84.48	1.99 [1.04;3.80]	0.04	137	77.40	45	84.91	1.64 [0.72;3.77]	0.24
BDI total score >15	66	57.89	83	74.77	2.16 [1.22;3.80]	0.008	104	60.12	43	86.00	4.07 [1.73;9.58]	0.001
Alcohol dependence or abuse	29	25.00	36	31.03	1.35 [0.76;2.40]	0.31	45	25.42	20	37.74	1.78 [0.93;3.41]	0.08
Substance dependence or abuse	19	16.38	25	21.55	1.40 [0.72;2.72]	0.32	33	18.64	11	20.75	1.14 [0.53;2.45]	0.73
Severe suicide attempt	24	20.69	20	17.24	0.80 [0.41;1.54]	0.50	33	18.64	11	20.75	1.14 [0.53;2.45]	0.73
Violent suicide attempt	12	10.34	16	13.79	1.39 [0.62;3.08]	0.42	25	14.12	3	5.66	0.36 [0.11;1.26]	0.11
Number of suicide attempts > 2	40	34.48	52	44.83	1.54 [0.91;2.62]	0.11	64	36.16	27	50.94	1.83 [0.99;3.41]	0.06

Continuous variables were expressed as medians (minimum value-maximum value)

Impulsivity, aggressiveness/hostility, anger, affect intensity and lability traits were significantly associated with PMS in a dose-response relationship (Table 2). These associations remained significant after subsequent adjustment for educational attainment, lifetime MDD, bipolar disorder, anxiety disorders, current depression and number of suicide attempts, except for cognitive impulsivity (BIS10) (p = 0.07) and suspicion (BDHI scale) (p = 0.16) (Table 2). In order to identify the personality traits best predicting PMS, significant traits associated with PMS in a univariate analysis were introduced into a multifactorial model adjusted for the same potential confounders. PMS was associated with trait anger (OR = 1.26, 95% CI = 0.52–3.06 for a score between 23–28 corresponding to the second tertile of the distribution; OR = 3.96, 95% CI = 1.42–11.0 for a score≥ 29 corresponding to the third tertile; p = 0.01) and affect lability (OR = 2.52, 95% CI = 1.06–5.96 for a score between 1.67 and 2.21 (second tertile); OR = 3.26 95% CI = 1.37–7.76 for a score ≥ 2.22 (third tertile), p = 0.02) independently of other personality traits (Table 3).

**Table 2:** Personality traits of patients according to PMS.

	PMS							
	PAF<115 N = 116		PAF≥115 N = 116		Model 0		Model 1	
	n	%	n	%	OR [95%CI]	p	OR [95%CI]	p
BIS10- total score								
<56	51	43.97	21	18.10	1	<0.0001	1	0.0004
[56–70[	37	31.90	36	31.03	2.36 [1.19;4.69]		2.22 [1.07;4.61]	
≥70	28	24.14	59	50.86	5.12 [2.60;10.1]		4.31 [2.08;8.94]	
BIS10—planning								
<17	44	37.93	25	21.55	1	0.001	1	0.005
[17–23[	43	37.07	36	31.03	1.47 [0.76;2.85]		1.53 [0.74;3.16]	
≥23	29	25.00	55	47.41	3.34 [1.72;6.50]		3.25 [1.57;6.71]	
BIS10—cognitive impulsivity								
<19	36	31.03	31	26.72	1	0.02	1	0.07
[19–25[	49	42.24	34	29.31	0.81 [0.42;1.54]		0.74 [0.37;1.49]	
≥25	31	26.72	51	43.97	1.91 [0.99;3.68]		1.62 [0.80;3.28]	
BIS10—motor impulsivity								
<17	53	45.69	21	18.10	1	<0.0001	1	<0.0001
[17–26[	38	32.76	41	35.34	2.72 [1.39;5.33]		2.71 [1.32;5.55]	
≥26	25	21.55	54	46.55	5.45 [2.73;10.9]		5.42 [2.51;11.7]	
BDHI								
<36	55	47.41	21	18.10	1	<0.0001	1	<0.0001
[36–44[	41	35.34	33	28.45	2.11 [1.07;4.16]		1.96 [0.96;3.98]	
≥44	20	17.24	62	53.45	8.12 [3.98;16.5]		6.41 [3.00;13.7]	
BDHI Assault								
≤2	55	47.41	31	26.72	1	0.0009	1	0.03
[3–5[	33	28.45	32	27.59	1.72 [0.89;3.31]		1.47 [0.73;2.96]	
≥5	28	24.14	53	45.69	3.36 [1.78;6.34]		2.57 [1.30;5.10]	
BDHI Indirect hostility								
<5	47	40.52	21	18.10	1	0.0007	1	0.005
[5–7[	40	34.48	47	40.52	2.63 [1.35;5.11]		2.48 [1.22;5.04]	
≥7	29	25.00	48	41.38	3.70 [1.86;7.39]		3.33 [1.57;7.05]	
BDHI Irritability								
<7	40	34.48	13	11.21	1	<0.0001	1	0.0008
[7–9[	37	31.90	37	31.90	3.08 [1.42;6.67]		2.95 [1.29;6.76]	
≥9	39	33.62	66	56.90	5.21 [2.48;10.9]		4.84 [2.13;11.0]	
BDHI negativism								
<2	19	16.38	10	8.62	1	0.001	1	0.01
[2–4[	63	54.31	45	38.79	1.36 [0.58;3.19]		0.98 [0.39;2.42]	
≥4	34	29.31	61	52.59	3.41 [1.42;8.16]		2.37 [0.94;5.93]	
BDHI resentment								
≤5	66	56.90	35	30.17	1	0.0001	1	
6	24	20.69	28	24.14	2.20 [1.11;4.35]		1.56 [0.74;3.29]	0.02
≥7	26	22.41	53	45.69	3.84 [2.06;7.17]		2.72 [1.34;5.50]	
BDHI suspicion								
<5	41	35.34	23	19.83	1	0.02	1	0.16
[5–7[	36	31.03	38	32.76	1.88 [0.95;3.73]		1.64 [0.78;3.43]	
≥7	39	33.62	55	47.41	2.51 [1.31;4.84]		1.98 [0.98;3.99]	
BDHI hostility								
<7	39	33.62	25	21.55	1	0.008	1	0.01
[7–9[	41	35.34	32	27.59	1.22 [0.62;2.41]		1.39 [0.67;2.90]	
≥9	36	31.03	59	50.86	2.56 [1.33;4.90]		2.79 [1.37;5.67]	
BDHI guilt								
<6	32	27.59	16	13.79	1	0.0007	1	0.004
[6–8[	48	41.38	36	31.03	1.50 [0.72;3.14]		1.33 [0.60;2.98]	
≥8	36	31.03	64	55.17	3.56 [1.72;7.35]		3.22 [1.44;7.21]	
STAXI trait anger								
<23	59	50.86	16	13.79	1	<0.0001	1	<0.0001
[23–29[	40	34.48	38	32.76	3.50 [1.72;7.12]		2.41 [1.14;5.11]	
≥29	17	14.66	62	53.45	13.45 [6.23;29.0]		10.80 [4.84;24.1]	
H scale								
<9	40	34.48	37	31.90	1	0.55	1	0.46
[9–13[	33	28.45	28	24.14	0.92 [0.47;1.80]		0.70 [0.32;1.51]	
≥13	43	37.07	51	43.97	1.28 [0.70;2.35]		0.62 [0.29;1.34]	
ALS								
<1.67	60	51.72	17	14.66	1	<0.0001	1	<0.0001
[1.67–2.22[	33	28.45	44	37.93	4.71 [2.33;9.50]		4.00 [1.88;8.52]	
≥2.22	23	19.83	55	47.41	8.44 [4.08;17.4]		5.70 [2.64;12.3]	
AIM								
<3.8	52	44.83	24	20.69	1	0.0006	1	0.004
[3.8–4.25[	33	28.45	44	37.93	2.89 [1.49;5.60]		2.79 [1.37;5.69]	
≥4.25	31	26.72	48	41.38	3.35 [1.73;6.50]		3.02 [1.47;6.19]	

Model 0: Crude associations

Model 1: Adjustment for educational level, major depressive disorder, bipolar disorder, anxiety disorder, total BDI score and number of suicide attempts.

AIM = Affect Intensity Measure Scale; ALS = Affect Lability Scale; BDHI = Buss-Durkee Hostility Scale; BIS = Barratt Impulsivity Scale; H Scale = Beck Hopelessness Scale; PAF = Premenstrual Assessment Form; PMS = Premenstrual Syndrome; STAXI = Spielberger State-Trait Anger Expression Inventory

**Table 3:** Personality traits of patients according to PMDD.

	PMDD							
	No N = 177		Yes N = 53		Model 0		Model 1	
	n	%	n	%	OR [95%CI]	p	OR [95%CI]	p
BIS10- total score								
<56	62	35.03	10	18.87	1	0.006	1	0.05
[56–70[	58	32.77	13	24.53	1.39 [0.57;3.41]		1.24 [0.46;3.36]	
≥70	57	32.20	30	56.60	3.26 [1.46;7.27]		2.70 [1.08;6.75]	
BIS10—planning								
<17	58	32.77	11	20.75	1	0.04	1	0.11
[17–23[	63	35.59	15	28.30	1.26 [0.53;2.95]		1.35 [0.52;3.52]	
≥23	56	31.64	27	50.94	2.54 [1.15;5.61]		2.43 [0.99;5.97]	
BIS10—cognitive impulsivity								
<19	54	30.51	13	24.53	1	0.009	1	0.01
[19–25[	70	39.55	12	22.64	0.71 [0.30;1.68]		0.71 [0.27;1.82]	
≥25	53	29.94	28	52.83	2.19 [1.03;4.69]		2.36 [1.00;5.58]	
BIS10—motor impulsivity								
<17	63	35.59	9	16.98	1	0.004	1	0.04
[17–26[	63	35.59	16	30.19	1.78 [0.73;4.32]		1.43 [0.55;3.74]	
≥26	51	28.81	28	52.83	3.84 [1.66;8.87]		2.94 [1.19;7.27]	
BDHI								
<36	67	37.85	9	16.98	1	<0.0001	1	0.01
[36–44[	62	35.03	12	22.64	1.44 [0.57;3.65]		1.36 [0.51;3.59]	
≥44	48	27.12	32	60.38	4.96 [2.17;11.3]		3.35 [1.38;8.14]	
BDHI Assault								
≤2	73	41.24	13	24.53	1	0.05	1	0.33
[3–5[	49	27.68	15	28.30	1.72 [0.75;3.93]		1.28 [0.53;3.10]	
≥5	55	31.07	25	47.17	2.55 [1.20;5.44]		1.88 [0.81;4.35]	
BDHI Indirect hostility								
<5	59	33.33	9	16.98	1	0.07	1	0.27
[5–7[	65	36.72	22	41.51	2.22 [0.95;5.20]		1.68 [0.68;4.16]	
≥7	53	29.94	22	41.51	2.72 [1.15;6.43]		2.17 [0.84;5.56]	
BDHI Irritability								
<7	51	28.81	2	3.77	1	0.0003	1	0.004
[7–9[	60	33.90	14	26.42	5.95 [1.29;27.4]		4.76 [0.98;23.1]	
≥9	66	37.29	37	69.81	14.29 [3.29;62.1]		10.24 [2.22;47.3]	
BDHI negativism								
<2	26	14.69	3	5.66	1	0.004	1	0.04
[2–4[	90	50.85	18	33.96	1.73 [0.47;6.34]		1.48 [0.39;5.71]	
≥4	61	34.46	32	60.38	4.55 [1.28;16.2]		3.50 [0.90;13.5]	
BDHI resentment								
≤5	91	51.41	9	16.98	1	<0.0001	1	0.004
6	41	23.16	11	20.75	2.71 [1.04;7.05]		1.86 [0.67;5.16]	
≥7	45	25.42	33	62.26	7.41 [3.27;16.8]		4.28 [1.75;10.5]	
BDHI suspicion								
<5	56	31.64	8	15.09	1	0.06	1	0.38
[5–7[	55	31.07	18	33.96	2.29 [0.92;5.70]		1.94 [0.74;5.12]	
≥7	66	37.29	27	50.94	2.86 [1.20;6.81]		1.72 [0.68;4.37]	
BDHI hostility								
<7	52	29.38	12	22.64	1	0.03	1	0.06
[7–9[	61	34.46	11	20.75	0.78 [0.32;1.92]		0.80 [0.31;2.11]	
≥9	64	36.16	30	56.60	2.03 [0.95;4.36]		2.04 [0.89;4.64]	
BDHI guilt								
<6	40	22.60	8	15.09	1	0.08	1	0.14
[6–8[	68	38.42	15	28.30	1.10 [0.43;2.83]		0.63 [0.22;1.79]	
≥8	69	38.98	30	56.60	2.17 [0.91;5.20]		1.42 [0.54;3.70]	
STAXI trait anger								
<23	72	40.68	3	5.66	1	<0.0001	1	0.0002
[23–29[	61	34.46	17	32.08	6.69 [1.87;23.9]		5.20 [1.41;19.3]	
≥29	44	24.86	33	62.26	17.99 [5.21;62.2]		12.94 [3.61;46.3]	
H scale								
<9	66	37.29	11	20.75	1	0.008	1	0.31
[9–13[	50	28.25	11	20.75	1.32 [0.53;3.29]		1.20 [0.44;3.27]	
≥13	61	34.46	31	58.49	3.05 [1.41;6.59]		1.94 [0.77;4.92]	
ALS								
<1.67	72	40.68	5	9.43	1	0.0006	1	0.01
[1.67–2.22[	54	30.51	22	41.51	5.87 [2.09;16.5]		3.74 [1.25;11.2]	
≥2.22	51	28.81	26	49.06	7.34 [2.64;20.4]		4.87 [1.68;14.1]	
AIM								
<3.8	66	37.29	9	16.98	1	0.02	1	0.03
[3.8–4.25[	57	32.20	19	35.85	2.44 [1.03;5.83]		2.54 [1.00;6.47]	
≥4.25	54	30.51	25	47.17	3.39 [1.46;7.88]		3.48 [1.40;8.67]	

Model 0: Crude associations

Model 1: Adjustment for age, study level, total BDI score, alcohol dependence or abuse, number of suicide attempts and violent suicide attempt. AIM = Affect Intensity Measure Scale; ALS = Affect Lability Scale; BDHI = Buss-Durkee Hostility Scale; BIS = Barratt Impulsivity Scale; H Scale = Beck Hopelessness Scale; PAF = Premenstrual Assessment Form; PMS = Premenstrual Syndrome; STAXI = Spielberger State-Trait Anger Expression Inventory

### 4.3. Personality traits and PMDD

Baseline characteristics of patients according to the presence of PMDD are listed in Table 1. Patients with PMDD were more likely to have a low educational attainment (p = 0.04), and severe current depression (p = 0.001) than patients without PMDD. Subsequent analyses were thus adjusted for these factors, and for age (p = 0.07), alcohol dependence/abuse (p = 0.08), violent suicide attempt (p = 0.11), and number of suicide attempts (p = 0.06). Other demographic covariates (marital status, present occupation, hormonal birth control) and psychiatric comorbidities (lifetime unipolar and bipolar disorder, anxiety disorder, substance use disorder and tobacco use, severity of suicide attempts) were not significantly associated with PMDD. In regards to the different phases of the menstrual cycle, no significant difference was evidenced between women with PMDD and those without (p = 0.53).

Impulsivity, anger, hopelessness, affect intensity and lability traits were significantly associated with PMDD in a dose-response relationship. Aggressiveness/hostility traits were also associated with PMDD (BDHI scale), except for assault, indirect hostility, suspicion and guilt dimensions (p = 0.05, p = 0.07, p = 0.06, p = 0.08 respectively) (Table 3). These associations remained significant after subsequent adjustment for educational attainment, current depression, age, alcohol dependence/abuse, violent suicide attempt and number of suicide attempts, except for the non-planning dimension of impulsivity (BIS10 scale) (p = 0.11), the hostility dimension (BDHI scale) (p = 0.06) and hopelessness (p = 0.31) (Table 2). In the common multifactorial analyses, PMDD was associated with trait anger independently of other personality traits (OR = 4.28, 95% CI = 1.00–18.4 for a score between 23 and 28; OR = 9.20, 95% CI = 1.98–42.8 for a score ≥ 29; p = 0.01) (Table 4).

**Table 4:** Common analysis of the associations between personality traits and PMS and PMDD.

	PAF≥115 vs PAF<115		PMDD ns no PMDD	
	OR [95%CI]^(1)^	p^(1)^	OR [95%CI]^(2)^	p^(2)^
BIS10- Total score				
<56	1	0.24	1	0.51
[56–70[	1.49 [0.65;3.46]		0.67 [0.22;2.05]	
≥70	2.09 [0.88;4.93]		1.13 [0.39;3.28]	
BDHI—Total score				
<36	1	0.28	1	0.62
[36–44[	1.09 [0.47;2.52]		0.59 [0.19;1.81]	
≥44	2.00 [0.77;5.18]		0.79 [0.25;2.46]	
STAXI—Trait anger				
<23	1	0.01	1	0.01
[23–29[	1.26 [0.52;3.06]		4.28 [1.00;18.4]	
≥29	3.96 [1.42;11.0]		9.20 [1.98;42.8]	
ALS–Total score				
<1.67	1	0.02	1	0.37
[1.67–2.22[	2.52 [1.06;5.96]		2.16 [0.66;7.12]	
≥2.22	3.26 [1.37;7.76]		2.28 [0.69;7.54]	
AIM–Total score				
<3.8	1	0.44	1	0.50
[3.8–4.25[	1.73 [0.75;4.00]		1.44 [0.51;4.11]	
≥4.25	1.37 [0.59;3.21]		1.84 [0.66;5.12]	

^(1)^ All personality traits associated with PMS at p<0.15 were included in the model and were adjusted for study level, anxiety disorders, major depressive disorder, bipolar disorder, number of suicide attempts and total BDI score.

^(2)^ All personality traits associated with PMDD at p<0.15 were included in the model and were adjusted for age, study level, total BDI score, alcohol dependence or abuse, number of suicide attempts and violent suicide attempt.

AIM = Affect Intensity Measure Scale; ALS = Affect Lability Scale; BDHI = Buss-Durkee Hostility Scale; BIS = Barratt Impulsivity Scale; STAXI = Spielberger State-Trait Anger Expression Inventory

Among patients with severe PMS, 60.5% also had PMDD and for patients with PMDD, 86.8% experienced severe PMS.

## Discussion

In this study, we examined the association between personality traits involved in suicidal vulnerability and premenstrual disturbances. In our sample, all women—those with and without PMS/PMDD—had attempted suicide Women with PMS or PMDD were more likely to exhibit most of these personality traits vs. those without, even after controlling for potential confounders, and independently from the phases of the menstrual cycle. Thus, female attempters with PMS or PMDD were more impulsive, aggressive and hostile, and this consistently across most dimensions or subscales of the assessment tools, and they also scored higher in instability and intensity of their emotional experience. Notably, we found impulsive-aggressive and emotional dysregulation patterns of personality in both women with PMS and women with PMDD, which are putative endophenotypes associated with vulnerability to suicidal behavior [46, 47]. Only trait anger remained associated with both PMS and PMDD independently of all other personality traits. This is interesting because aggressiveness—the behavioral expression of anger—seems to be the trait that differentiates depressed suicide attempters from non-attempters, rather than global personality dimensions such as impulsiveness or hostility [48]. Indeed, trait anger distinguishes prisoners with a history of suicide attempts from those with not prior history of SA [49]. Moreover, the suicide risk profile was previously associated with personality features. Indeed, in a previous study based on a partially overlapping sample, higher levels of trait anger were also linked to major suicide attempt repeaters (individuals with ≥ 5 lifetime suicide attempts) [48]. Furthermore, higher levels of trait anger were associated with violent suicide attempts [50]. Similarly, the lethality of the suicide attempts in borderline patients resulted from an interaction between impulsivity and violent-aggressive features [51]. However, in the present study, women with PMS/PMDD did no exhibit a more severe suicide risk profile (frequent, severe and violent) than women without PMS/PMDD. Thus, the present study cannot support the existence of a more severe risk profile in women with both PMS/PMDD and high trait-anger level. On the other hand, the high frequency of PMS/PMDD among attempters (50% and 23% respectively) highlights the need to assess the premenstrual symptomatology of suicide attempters in daily clinical practice. Within this evaluation, questioning patients about anger management could provide an important clue to a potential risk of suicide attempt.

Women with high anger levels were four times more likely to suffer from PMS, and nine times more likely to suffer from PMDD than women with low anger levels. Moreover, the higher the anger level, the greater the risk to present with PMS and PMDD. The association of anger and PMS/PMDD is in line with previous studies reporting higher trait anger in women with PMDD compared with healthy controls [52–54]. Interestingly, higher anger levels were associated with decreased life satisfaction [55], and psychosocial vulnerability (with decreased positive (i.e. support) relationships and increased negative (i.e. conflict) ones [56, 57].

Furthermore, women with PMS/PMDD do not typically present with hormonal imbalance; rather, they appear to be particularly sensitive to the normal fluctuations of the gonadal steroids, estrogen and progesterone, occurring with each menstrual cycle [58]. This observed sensitivity may be partially explained by the potent central nervous system effects of the neuroactive metabolites of progesterone, allopregnanolone, and pregnanolone. ALLO and pregnanolone are positive modulators of the gamma-aminobutyric acid (GABA) inhibitory neurotransmitter system. In fact, ALLO and pregnanolone, neuroactive derivatives of progesterone, are able to modulate the subunit component of the GABA-A receptor [59], thus altering the function of this receptor. Lower levels of peripheral ALLO were measured in women with PMS/PMDD during the luteal phase, and women with PMDD in the luteal phase showed a blunted ALLO stress response compared to non-PMDD controls. These results promoted the hypothesis that stress-induced dysregulation of ALLO may contribute to the pathogenesis of PMS/PMDD [60–64]. Measurement of cortical excitability using transcranial magnetic stimulation (TMS) demonstrated that women with PMDD showed increased facilitation in the luteal phase compared to controls [65], indicating an alteration in balance between cortical excitation and inhibition. Decreased GABA levels result in a decreased tonic inhibition [66, 67], which in turn leads to an increased metabolic activity [68], which therefore might produce more glutamate. Thus, the GABA neurotransmitter system dysregulation seems to be involved in PMDD [2, 69] and in suicidal behavior [70]. Interestingly, Liu et al [71] found that GABA levels were reduced in the Anterior Cingulate Cortex/medial Prefrontal Cortex (ACC/mPFC) regions in PMDD women as compared with healthy controls. Finally, a recent fMRI study provided evidence on the impact of decreased ACC GABA levels in patients with high trait anger levels [72]. Thus, decreased ACC GABA levels seem to be a common pattern for both suicidal behavior and PMS/PMDD involving high trait anger levels.

Selective serotonin reuptake inhibitors (SSRIs), currently the first-line therapy for PMS/PMDD [73], increase brain GABA levels [74, 75]. Indeed, the serotonin system plays an important role in both PMS/PMDD and suicidal behavior [76]. Saunders and Hawton suggested that lower levels of serotonin present in the low estrogen (luteal) phases of the cycle may increase the likelihood of suicidal behavior [77]. Moreover, in the premenstrual phase, women with PMDD had lower whole blood serotonin concentrations and decreased platelet serotonin reuptake compared to women without PMDD [78, 79]. Interestingly, several studies linked low serotonin levels and aggressiveness: i) concentration levels of serotonin (5-HT) and its main metabolite, 5-hydoxyindoleacetic acid (5-HIAA), were lower in aggressive subjects vs. non-aggressive subjects [80]; ii) reduced CSF concentration of 5-HIAA was observed in individuals with a history of aggression [81], impulsive aggression [82] and higher level of trait anger [83]; iii) there is also strong evidence pointing to the involvement of the serotonin system in suicidal behavior. For instance, suicide attempters, especially those using violent methods, exhibited lower CSF 5-HIAA levels compared to psychiatric controls, and those taking subsequent suicidal actions also exhibited lower CSF 5-HIAA levels [70, 84]. As a matter of fact, it was suggested that the well-documented association between suicide and the serotonin system was mediated by aggression dyscontrol [85].

To continue, data consistently show that elevated trait anger expressiveness (anger-out) is associated with increased response to acute and chronic pain (CP) [86–90]. In fact, pain is a common symptom in PMS/PMDD [91], possibly associated with variability in testosterone (and to a lesser degree estradiol) [92], serotonergic [93, 94] and endogenous opioidergic [95–97] dysfunctions. Of note, previous studies found higher levels of hyperalgesia in women with PMDD, independently of the phases of the menstrual cycle [91, 92, 97, 98]. On the basis of the ironic process theory [99], Quartana et al [100] found that attempts to suppress anger may amplify pain sensitivity by paradoxically increasing the perception of pain-related irritating and frustrating qualities. It is also quite interesting since experiential avoidance (tendency to escape or avoid unwanted thoughts, emotions, memories, and sensations [99, 101] was shown to be predictive of suicidal behaviors [102, 103].

Our present results are relevant for therapeutic perspectives in PMS/PMDD. In fact, mindfulness-based therapies are psychological interventions based on decreased experiential avoidance, thus increased psychological flexibility [101]. The third wave of cognitive behavioral therapies showed improvement in trait anger as compared with patients on a waiting list [86, 104], as well as improved pain management [105, 106] and suicidal patients [107, 108]. Thus, these psychotherapies seem to represent a promising adjuvant treatment for managing PMS/PMDD patients.

The first limitation of this study is that cross-sectional data cannot lead us to conclude to a causal or direct relationship between PMS/PMDD and personality of suicide attempters. Thus, further longitudinal studies are needed to test this hypothesis. Secondly, the type of tool used for assessing personality traits (self-report questionnaire) and PAF (retrospective assessment, but only focusing on the last three menstrual cycles) might also represent a limit. Furthermore, the assessment of PMS and PMDD with the PAF also shows limitations as the PAF covers 14 days–not one week as defined in the DSM-5 diagnosis of PMDD–prior to the onset of menses. However, all questionnaires included in this study were previously validated and are commonly used in clinical practice. Of note, a recall bias may affect the responses of women that were not in a premenstrual phase at the time of the evaluation. Finally, either DIGS or MINI were used depending on the level of suicidality but no data is available to evaluate the concordance between these instruments. However, the inter-rater and test-retest reliability of major mood and psychotic diagnoses in the DIGS have been established for psychiatric patients (overall kappa coefficient = 0.87 and 0.60 respectively) [42]. Besides, our study was conducted in a sample of women who all attempted suicide, in order to assess the mechanisms underlying the association between suicidal behaviors and PMS/PMDD, such as personality features. Thus, we have to keep in mind that the present sample is not a representative sample of all women with or without PMS/PMDD. Besides, PMDD status was assessed using DSM 5 items from the PAF-shortened form that amounted to a simple score that validated the diagnosis. We are the first team to use this method. In our sample, among patients with severe PMS, only 60.5% had also PMDD. This difference could be explained by the absence of mood symptoms in some of the women with severe PMS. Inversely, 87% of the patients with PMDD experienced also severe PMS. PMDD diagnostic criteria imply also a PMS diagnosis, but symptoms might not be intense enough (i.e. less than extreme scores in the PAF) to fulfill severe PMS diagnosis. Finally, there is a persisting debate in the scientific community whether PMDD is in fact a distinct clinical entity [109].

In conclusion, this study demonstrates a strong, independent association between PMS/PMDD and trait anger in a representative sample of female suicide attempters. It is of major interest for clinicians in view of addressing a substantial public health problem among women of reproductive age. Finally, the latest innovating psychological interventions involving mindfulness should be tested in this population.

## Supporting Information

S1 Supplementary Material Diagnostic criteria for PMDD according to DSM5, and algorithm used to assess PMDD status using the PAF-shortened form. *To calculate PMDD diagnoses with the PAF we followed the DSM criteria as closely as possible*. *Criterion A was roughly fulfilled by the introductory formulation of the PAF*. *PMDD diagnoses included at least one symptom of extreme severity corresponding to Criterion B and one symptom of extreme severity corresponding to Criterion C*. *In total*, *at least five symptoms of extreme severity listed in Criteria B or C were endorsed*. *The use of extreme severity as a cutoff for these symptoms provided evidence that a clinically significant distress or interference with daily activities (Criterion D) was present*. *Criteria E*, *F and G could not be assessed*. A. In the majority of menstrual cycles, at least five symptoms must be present in the final week before the onset of menses, they should start to improve within a few days after the onset of menses, and become minimal or absent in the week post menses. B. One (or more) of the following symptoms must be present: 1. Marked affective lability (e.g., mood swings: feeling suddenly sad or tearful, or increased sensitivity to rejection). *= > items 1*, *12 or 31 from PAF-shortened form*, *extreme score (6)* 2. Marked irritability or anger or increased interpersonal conflicts. *= > items 9*, *17or 20 from PAF-shortened form*, *extreme score (6)* 3. Marked depressed mood, feelings of hopelessness, or self-deprecating thoughts. *= > items 8 and 13 from PAF-shortened form*, *extreme score (6)* 4. Marked anxiety, tension, and/or feelings of being keyed up or on edge. *= > items 3 or 15 from PAF-shortened form*, *extreme score (6)* C. One (or more) of the following symptoms must additionally be present, to reach a total of five symptoms when combined with symptoms from Criterion B above. 1. Decreased interest in usual activities (e.g., work, school, friends, hobbies). *= > items 23*, *24*, *27 or 33 from PAF-shortened form*, *extreme score (6)* 2. Subjective difficulty in concentration. *= > item 11 from PAF-shortened form*, *extreme score (6)* 3. Lethargy, easy fatigability, or marked lack of energy. *= > item 2 or 32 from PAF-shortened form*, *extreme score (6)* 4. Marked change in appetite; overeating; or specific food cravings. *= > item 20 from PAF-shortened form*, *extreme score (6)* 5. Hypersomnia or insomnia. *= > item 10 from PAF-shortened form*, *extreme score (6)* 6. A sense of being ovenwhelmed or out of control. *= > item 7 from PAF-shortened form*, *extreme score (6)* 7. Physical symptoms such as breast tenderness or swelling, joint or muscle pain, a sensation of “bloating,” or weight gain. *= > item 6*, *18 or 25 from PAF-shortened form*, *extreme score (6)* D. The symptoms are associated with clinically significant distress or interference with work, school, usual social activities, or relationships with others (e.g., avoidance of social activities; decreased productivity and efficiency at work, school, or home). E. The disturbance is not merely an exacerbation of the symptoms of another disorder, such as major depressive disorder, panic disorder, persistent depressive disorder (dysthymia), or a personality disorder (although it may co-occur with any of these disorders). F. Criterion A should be confirmed by prospective daily ratings during at least two symptomatic cycles. (Note: The diagnosis may be made provisionally prior to this confirmation.) G. The symptoms are not attributable to the physiological effects of a substance (e.g., an illicit substance or drug, a medication, other treatment) or another medical condition (e.g., hyperthyroidism).
(DOCX)
