# Supplementary material for: Personality Traits of Suicidality Are Associated with Premenstrual Syndrome and Premenstrual Dysphoric Disorder in a Suicidal Women Sample
Source: PLoS One. 2016 Feb 10;11(2):e0148653. doi: 10.1371/journal.pone.0148653 (PMC4749223; doi:10.1371/journal.pone.0148653)
Supplement: S1 Supplementary Material — To calculate PMDD diagnoses with the PAF we followed the DSM criteria as closely as possible. Criterion A was roughly fulfilled by the introductory formulation of the PAF. PMDD diagnoses included at least one symptom of extreme severity corresponding to Criterion B and one symptom of extreme severity corresponding to Criterion C. In total, at least five symptoms of extreme severity listed in Criteria B or C were endorsed. The use of extreme severity as a cutoff for these symptoms provided evidence that a clinically significant distress or interference with daily activities (Criterion D) was present. Criteria E, F and G could not be assessed. A. In the majority of menstrual cycles, at least five symptoms must be present in the final week before the onset of menses, they should start to improve within a few days after the onset of menses, and become minimal or absent in the week post menses. B. One (or more) of the following symptoms must be present: 1. Marked affective lability (e.g., mood swings: feeling suddenly sad or tearful, or increased sensitivity to rejection). = > items 1, 12 or 31 from PAF-shortened form, extreme score (6) 2. Marked irritability or anger or increased interpersonal conflicts. = > items 9, 17or 20 from PAF-shortened form, extreme score (6) 3. Marked depressed mood, feelings of hopelessness, or self-deprecating thoughts. = > items 8 and 13 from PAF-shortened form, extreme score (6) 4. Marked anxiety, tension, and/or feelings of being keyed up or on edge. = > items 3 or 15 from PAF-shortened form, extreme score (6) C. One (or more) of the following symptoms must additionally be present, to reach a total of five symptoms when combined with symptoms from Criterion B above. 1. Decreased interest in usual activities (e.g., work, school, friends, hobbies). = > items 23, 24, 27 or 33 from PAF-shortened form, extreme score (6) 2. Subjective difficulty in concentration. = > item 11 from PAF-shortened form, extreme score (6) 3. Lethargy, easy fatigabili [file pone.0148653.s001.docx]

S1. Supplementary material.

Diagnostic criteria for PMDD according to DSM5. The algorithm to assess PMDD status using the PAF-shortened form is detailed *in italics*.

*To calculate PMDD diagnoses with the PAF we followed the DSM criteria as closely as possible. Criterion A was roughly fulfilled by the introductory formulation of the PAF. PMDD diagnoses included at least one symptom of extreme severity corresponding to Criterion B and one symptom of extreme severity corresponding to Criterion C. In total, at least five symptoms of extreme severity listed in Criteria B or C were endorsed. The use of extreme severity as a cutoff for these symptoms provided evidence that a clinically significant distress or interference with daily activities (Criterion D) was present. Criteria E, F and G could not be assessed.*

A. In the majority of menstrual cycles, at least five symptoms must be present in the final week before the onset of menses, they should start to improve within a few days after the onset of menses, and become minimal or absent in the week post menses.

B. One (or more) of the following symptoms must be present:

1. Marked affective lability (e.g., mood swings: feeling suddenly sad or tearful, or increased sensitivity to rejection).

*=> items 1, 12 or 31 from PAF-shortened form, extreme score (6)*

2. Marked irritability or anger or increased interpersonal conflicts.

*=> items 9, 17or 20 from PAF-shortened form, extreme score (6)*

3. Marked depressed mood, feelings of hopelessness, or self-deprecating thoughts.

*=> items 8 and 13 from PAF-shortened form, extreme score (6)*

4. Marked anxiety, tension, and/or feelings of being keyed up or on edge.

*=> items 3 or 15 from PAF-shortened form, extreme score (6)*

C. One (or more) of the following symptoms must additionally be present, to reach a total of five symptoms when combined with symptoms from Criterion B above.

1. Decreased interest in usual activities (e.g., work, school, friends, hobbies).

*=> items 23, 24, 27 or 33 from PAF-shortened form, extreme score (6)*

2. Subjective difficulty in concentration.

*=> item 11 from PAF-shortened form, extreme score (6)*

3. Lethargy, easy fatigability, or marked lack of energy.

*=> item 2 or 32 from PAF-shortened form, extreme score (6)*

4. Marked change in appetite; overeating; or specific food cravings.

*=> item 20 from PAF-shortened form, extreme score (6)*

5. Hypersomnia or insomnia.

*=> item 10 from PAF-shortened form, extreme score (6)*

6. A sense of being ovenwhelmed or out of control.

*=> item 7 from PAF-shortened form, extreme score (6)*

7. Physical symptoms such as breast tenderness or swelling, joint or muscle pain, a sensation of “bloating,” or weight gain.

*=> item 6, 18 or 25 from PAF-shortened form, extreme score (6)*

D. The symptoms are associated with clinically significant distress or interference with work, school, usual social activities, or relationships with others (e.g., avoidance of social activities; decreased productivity and efficiency at work, school, or home).

E. The disturbance is not merely an exacerbation of the symptoms of another disorder, such as major depressive disorder, panic disorder, persistent depressive disorder (dysthymia), or a personality disorder (although it may co-occur with any of these disorders).

F. Criterion A should be confirmed by prospective daily ratings during at least two symptomatic cycles. (Note: The diagnosis may be made provisionally prior to this confirmation.)

G. The symptoms are not attributable to the physiological effects of a substance (e.g., an illicit substance or drug, a medication, other treatment) or another medical condition (e.g., hyperthyroidism).
